# RETRACTION: The Effect of Primary Closure Versus Secondary Closure Techniques on Postoperative Wound Pain in Patients Undergoing Mandibular Surgery: A Meta‐Analysis

**DOI:** 10.1111/iwj.70402

**Published:** 2025-03-21

## Abstract

**RETRACTION:** ZhangL. and LiL. , “The Effect of Primary Closure Versus Secondary Closure Techniques on Postoperative Wound Pain in Patients Undergoing Mandibular Surgery: A Meta‐Analysis,” International Wound Journal 21, no. 4 (2024): e14753, 10.1111/iwj.14753.38531356 PMC10965271

The above article, published online on 26 March 2024, in Wiley Online Library (http://onlinelibrary.wiley.com/), has been retracted by agreement between the journal Editor in Chief, Professor Keith Harding; and John Wiley & Sons Ltd. Following an investigation by the publisher, all parties have concluded that this article was accepted solely on the basis of a compromised peer review process. The editors have therefore decided to retract the article. The authors did not respond to our notice regarding the retraction.

**RETRACTION:** L. Zhang and L. Li , “The Effect of Primary Closure Versus Secondary Closure Techniques on Postoperative Wound Pain in Patients Undergoing Mandibular Surgery: A Meta‐Analysis,” International Wound Journal 21, no. 4 (2024): e14753, 10.1111/iwj.14753.38531356 PMC10965271

The above article, published online on 26 March 2024, in Wiley Online Library (http://onlinelibrary.wiley.com/), has been retracted by agreement between the journal Editor in Chief, Professor Keith Harding; and John Wiley & Sons Ltd. Following an investigation by the publisher, all parties have concluded that this article was accepted solely on the basis of a compromised peer review process. The editors have therefore decided to retract the article. The authors did not respond to our notice regarding the retraction.

